# 

**Published:** 1914-06-20

**Authors:** 


### CONTENTS.

PAGE

**The Failure of Incipient Insanity Bills** ... 309

**Working-Class Support and Representation...** 310

**Hospital and Institutional News...** ... ... 311

The Mount Vernon Hospital Litigation—Our Bed-Lifter Competition Prize Scheme—The Lister Ward at Glasgow—The Effect of Criticism on Boards of Guardians—The Impression Created by the Bath Guardians—A Veteran Medical Journalist—Charing Cross Hospital History—Tuberculosis Pavilions at Isolation Hospitals—A Question of Dual Control—The Dean of York Resigns his Chairmanship—The Policy of Shunning Publicity—Secretarial Changes at Worcester Infirmary—Institutional Education for the Mentally Defective—The Doctors' Wives Association—The Insecurity of the Medical Officer of Health—Hospital Risks of Infection—New Research Laboratories at the Infants' Hospital—The Amateur Alienist—Reminiscences of the Septic Epoch—Guardians' Subscriptions to Voluntary Institutions—A Hospital Secretary as Architect—The Object of the Pay-Ward—Prince Alexander of Teck's Successor—The National Health Laboratory—A Medical Statistician—The Islington Tuberculosis Scheme—This Week's Drug Market.

**Common Errors in Diagnosis** ... ... ... 317

The Chest: By J. E. SQUIRE, M.D., C.B., Consulting Physician to Mount Vernon Hospital.

[See over.]

### CONTENTS—(*continued*).

PAGE

**Synthetic Germs** ... ... ... ... ... 318

**The School Doctor and the "G. P."** ... ... 319
Medical Etiquette Tested by Practice.

**Round the World** ... ... ... ... ... 320

Fellow-Workers and their Doings.

**Reports on Hospitals of the United Kingdom**— 321
By SIR HENRY BURDETT, K.C.B., K.C.V.O.
The Epsom and Ewell Cottage Hospital.
Faversham Cottage Hospital.

**Buildings Contemplated and in Progress** ... 322

**The Newcastle Conference** ... ... ... ... 323
The Place and Personalities of the Week.

**The Institutional Library** ... ... ... ... 326
Scientific By-paths—The Practising Operator.

**Gifts and Bequests** ... ... ... ... ... 326

**National Insurance Act Developments** ... ... 327
The Question of Linked-up Illnesses.

**Institutional Needs** ... ... ... ... ... 330

**Doctors' Wills** ... ... ... ... ... ... 331

PAGE

**The Editor's Letter-Box** ... ... ... ... 329
The Tuberculosis Society—A Question of Professional Conduct—Institutional Defalcations: The Assistant Secretary's Responsibility—Hospital Advertising.

**Hospitals from the Patients' Point of View** ... 332
Three Weeks in a Pay-Ward.

**The Book Market** ... ... ... .. ... 333

**The Profession of Medicine** ... ... ... 334
The Council Election of the English Royal College of Surgeons.
A Novel Request.

**The Institutional Worker** ... ... (Suppl.) 1
Hospital Inventories.
Questions for June.
Questions Invited.
Hospital Supplies.
The Sick and in Need.
Acknowledgments.
Employment and Training.

